# A Systematic Review of Case Series and Clinical Trials Investigating Regenerative Medicine for the Treatment of Vitiligo

**DOI:** 10.1111/jocd.16660

**Published:** 2024-11-07

**Authors:** Alireza Jafarzadeh, Arash Pour Mohammad, Azadeh Goodarzi

**Affiliations:** ^1^ Department of Dermatology, Rasool Akram Medical Complex Clinical Research Development Center (RCRDC), School of Medicine Iran University of Medical Sciences (IUMS) Tehran Iran; ^2^ Faculty of Medicine Iran University of Medical Sciences Tehran Iran

**Keywords:** autologous melanocyte‐keratinocyte transplantation, platelet‐rich plasma, stromal vascular fraction, systematic review, vitiligo

## Abstract

**Aims and Objectives:**

The aim of this study is to examine the efficacy and safety of various regenerative medicine treatments, such as cell therapy, platelet‐rich plasma (PRP), plasma‐poor platelet (PPP), plasma‐rich fibrin (PRF), mesenchymal stem cells, stromal vascular fraction (SVF), exosomes, adipose‐derived stem cells (ADSC), and stem cell‐conditioned media (SC‐CM), for treating vitiligo.

**Method:**

We conducted a thorough search of major databases such as PubMed, Scopus, and Web of Science, and selected 48 articles based on specific criteria. We used EndNote X8 and Google Sheets to review and extract data from the articles. After analyzing the studies, we categorized them accordingly.

**Results:**

This systematic review analyzed 48 articles involving 2186 patients with vitiligo to assess the effectiveness of regenerative medicine treatments. Key findings revealed that methods such as autologous non‐cultured melanocyte‐keratinocyte transplantation and platelet‐rich plasma (PRP) injection exhibited significant repigmentation, particularly when combined with modalities like NB‐UVB phototherapy and laser treatments. Notably, the autologous melanocyte‐keratinocyte transplantation achieved over 50% repigmentation within 9 months, while PRP demonstrated an average repigmentation of 58.7%, especially effective with CO_2_ laser treatment. Hair follicle‐derived cell transplantation also showed impressive response rates, achieving good to excellent results in up to 93.8% of patients. Side effects were noted in 21 of 28 studies, primarily involving pain, with no serious adverse events reported. The risk of bias assessment indicated that 37.21% of studies were low risk, while 48.84% had high risks overall. These findings suggest that while regenerative medicine holds promise for vitiligo treatment, further clinical trials are necessary to explore additional methods like stromal vascular fraction and exosomes.

**Conclusion:**

We have concluded that regenerative medicine plays an effective role in the treatment of vitiligo lesions. Furthermore, this treatment method is safe and does not cause serious complications. It can be used alone or in combination with other methods for treating vitiligo. To advance the treatment of vitiligo, we recommend conducting clinical trials on the unexplored branches of regenerative medicine.

## 1 Introduction

Vitiligo is an autoimmune skin disease in which melanocytes are destroyed. The manifestation of the disease often appears as depigmented patches in various areas of the body, which can significantly impact a person's mental health and relationships. Although it is not a common skin disease, with a prevalence of less than 2%, it can have unpleasant effects on patient's appearance [1, 2, 3].

Over the years, several treatments have been proposed to treat vitiligo, but their success rates and recurrence rates can vary. Topical treatments include topical corticosteroids, topical calcineurin inhibitors, and Janus kinase pathway inhibitors, while systemic treatments include oral and injectable corticosteroids, cyclosporine, methotrexate, azathioprine, and Janus kinase inhibitors. Phototherapy with Narrowband Ultraviolet B (NB‐UVB) has also shown promising results in aiding patient recovery [2, 4, 5, 6].

Regenerative medicine holds a special place in modern medicine, and its utilization in medical science is ever‐increasing. Dermatologists have adopted regenerative medicine techniques to treat various diseases, such as vitiligo, androgenic alopecia, as well as various types of atrophic and hypertrophic scars. Furthermore, over time, the applications of this treatment method have expanded to include inflammatory skin diseases and external injuries involving the skin [7, 8, 9].

Examples of regenerative medicine in dermatology include cell therapy, stromal vascular fraction (SVF), platelet‐rich plasma (PRP), exosomes, adipose‐derived stem cells (ADSC), Stem cells‐conditioned media (SCs‐CM), and melanocyte‐keratinocyte transplant procedure (MKTP), among others. Although the mentioned treatment methods may differ in terms of performance details, they share a common goal: tissue regeneration and the restoration of lost skin cell [10, 11, 12, 13].

In recent years, clinical trials and case series studies have showcased the potential of regenerative medicine methods for treating vitiligo. Although the most important aspect of this treatment method in vitiligo lesions is related to melanocyte‐keratinocyte transplantation with different treatment methods, the expansion of other aspects of regenerative medicine in the treatment of vitiligo has made significant progress [10, 14, 15].

However, the lack of a comprehensive systematic study evaluating the effectiveness of these treatment approaches specifically for vitiligo lesions has prompted us to undertake the aforementioned study. Our objective is to conduct a systematic review of regenerative medicine treatments for vitiligo.

## 2 Methods and Materials

### 2.1 Search Strategy and Databases

We conducted a comprehensive search on major databases, including Medline (PubMed), Scopus, Embase, and Web of Science (ISI), on January 30, 2024. The search syntax was tailored for each database and included a range of regenerative treatments such as “Platelet‐Rich Plasma (PRP)”, “Stromal Vascular Fraction (SVF)”, “exosomes”, “adipose‐derived stem cells conditioned medium”, “fat transfer”, “nano fat”, “lipotransfer”, “fat grafting”, “stem cell”, “cell therapy”, “Adipose Derived Stem Cell (ADSC)”, “lipografting”, “melanocyte‐keratinocyte transplantation”, and “epidermal melanocyte transplantation”. These terms were combined with variations of “vitiligo”, and relevant MeSH terms were incorporated to enhance search results in PubMed. We also conducted a manual examination of references within the included articles to identify any missing records. The search strategies used across databases are summarized in Table 1.

**TABLE 1:** Search strategies.

PubMed	(“cell therapy”[Title/Abstract] OR “SVF”[Title/Abstract] OR “Stromal Vascular Fraction”[Title/Abstract] OR “PRP”[Title/Abstract] OR “Platelet‐Rich Plasma”[Title/Abstract] OR “exosome”[Title/Abstract] OR “adipose derived stem cells conditioned medium”[Title/Abstract] OR “fat transfer”[Title/Abstract] OR “nano fat”[Title/Abstract] OR “lipotransfer”[Title/Abstract] OR “fat grafting”[Title/Abstract] OR “stem cell therapy”[Title/Abstract] OR “stromal vascular fraction”[Title/Abstract] OR “extracellular vesicle”[Title/Abstract] OR “ADSC”[Title/Abstract] OR “Adipose Derived Stem Cell”[Title/Abstract] OR “platelet rich plasma”[Title/Abstract] OR OR “stem cell”[Title/Abstract] OR “lipografting”[Title/Abstract] OR “melanocyte keratinocyte transplantation”[Title/Abstract] OR “melanocyte‐keratinocyte transplantation”[Title/Abstract] OR “epidermal melanocyte transplantation”[Title/Abstract] OR “autologous melanocyte‐keratinocyte suspension”[Title/Abstract]) AND (“vitiligo”[Title/Abstract] OR “vitiligo”[MeSH Terms]) AND (2010:2024[pdat])
Scopus	(TITLE‐ABS‐KEY (“cell therapy” OR “SVF” OR “Stromal Vascular Fraction” OR “PRP” OR “Platelet‐Rich Plasma” OR “exosome” OR “adipose derived stem cells conditioned medium” OR “fat transfer” OR “nano fat” OR “lipotransfer” OR “fat grafting” OR “stem cell therapy” OR “stromal vascular fraction” OR “extracellular vesicle” OR “ADSC” OR “Adipose Derived Stem Cell” OR “platelet rich plasma” OR “platelet‐rich plasma” OR “stem cell” OR “lipografting” OR “melanocyte keratinocyte transplantation” OR “melanocyte‐keratinocyte transplantation” OR “epidermal melanocyte transplantation” OR “autologous melanocyte‐keratinocyte suspension”)) AND TITLE‐ABS‐KEY (“vitiligo”) AND PUBYEAR > 2009 AND PUBYEAR < 2025
Web of science	TS = (“Procedural treatment” OR “cell therapy” OR “SVF” OR “Stromal Vascular Fraction” OR “PRP” OR “Platelet‐Rich Plasma” OR “exosome” OR “adipose derived stem cells conditioned medium” OR “fat transfer” OR “nano fat” OR “lipotransfer” OR “fat grafting” OR “stem cell therapy” OR “stromal vascular fraction” OR “extracellular vesicle” OR “ADSC” OR “Adipose Derived Stem Cell” OR “platelet rich plasma” OR “platelet‐rich plasma” OR “stem cell” OR “lipografting” OR “melanocyte keratinocyte transplantation” OR “melanocyte‐keratinocyte transplantation” OR “epidermal melanocyte transplantation” OR “autologous melanocyte‐keratinocyte suspension”) AND TS = (“vitiligo”)

### 2.2 Inclusion and Exclusion Criteria

The inclusion criteria comprised clinical articles and case series written in English that explored the use of regenerative medicine methods (such as cell therapy, stromal vascular fraction (SVF), platelet‐rich plasma (PRP), plasma‐poor platelet (PPP), plasma‐rich fibrin (PRF), mesenchymal stem cells, exosomes, adipose‐derived stem cells (ADSC), and stem cell‐conditioned media (SCs‐CM)) for the treatment of vitiligo. Exclusion criteria included review articles, non‐human studies (including animal and laboratory studies), studies that combined regenerative medicine with other treatment methods without a control group, and articles not available in full text.

### 2.3 Study Selection and Data Extraction

Two reviewers independently assessed the titles and abstracts of the retrieved records based on the eligibility criteria. The studies were analyzed for characteristics, including author, year, study design, treatment method used, follow‐up intervals, and criteria for evaluating improvement. Participant characteristics, such as mean age, gender ratio, and sample size, were recorded. We extracted results related to healing of lesions, patient satisfaction, adverse effects, and safety from the studies. EndNote X8 and Google Sheets were used for article screening and data extraction. Two researchers conducted the data collection independently, resolving any disagreements with the assistance of a more experienced researcher (A.G.).

### 2.4 Risk of Bias Assessment

The risk of bias for each included study was evaluated using the Cochrane Risk of Bias 2.0 (RoB 2) tool, which assesses five key domains: bias arising from the randomization process (D1), bias due to deviations from intended interventions (D2), bias due to missing outcome data (D3), bias in the measurement of the outcome (D4), and bias in the selection of the reported result (D5). Each domain was rated as having “low risk,” “some concerns,” or “high risk” of bias.

To ensure a detailed assessment of study quality, we also considered the following criteria: clarity of inclusion and exclusion criteria, sample size justification, description of intervention protocols, blinding methods, and comprehensive reporting of outcomes and adverse effects. Two main investigators conducted the assessments independently (A.J. and A.P.M.), and any discrepancies were resolved through discussion with a third reviewer (A.G.). Based on the individual domain ratings, we categorized the overall risk of bias for each study.

## 3 Result

**FIGURE 1:**
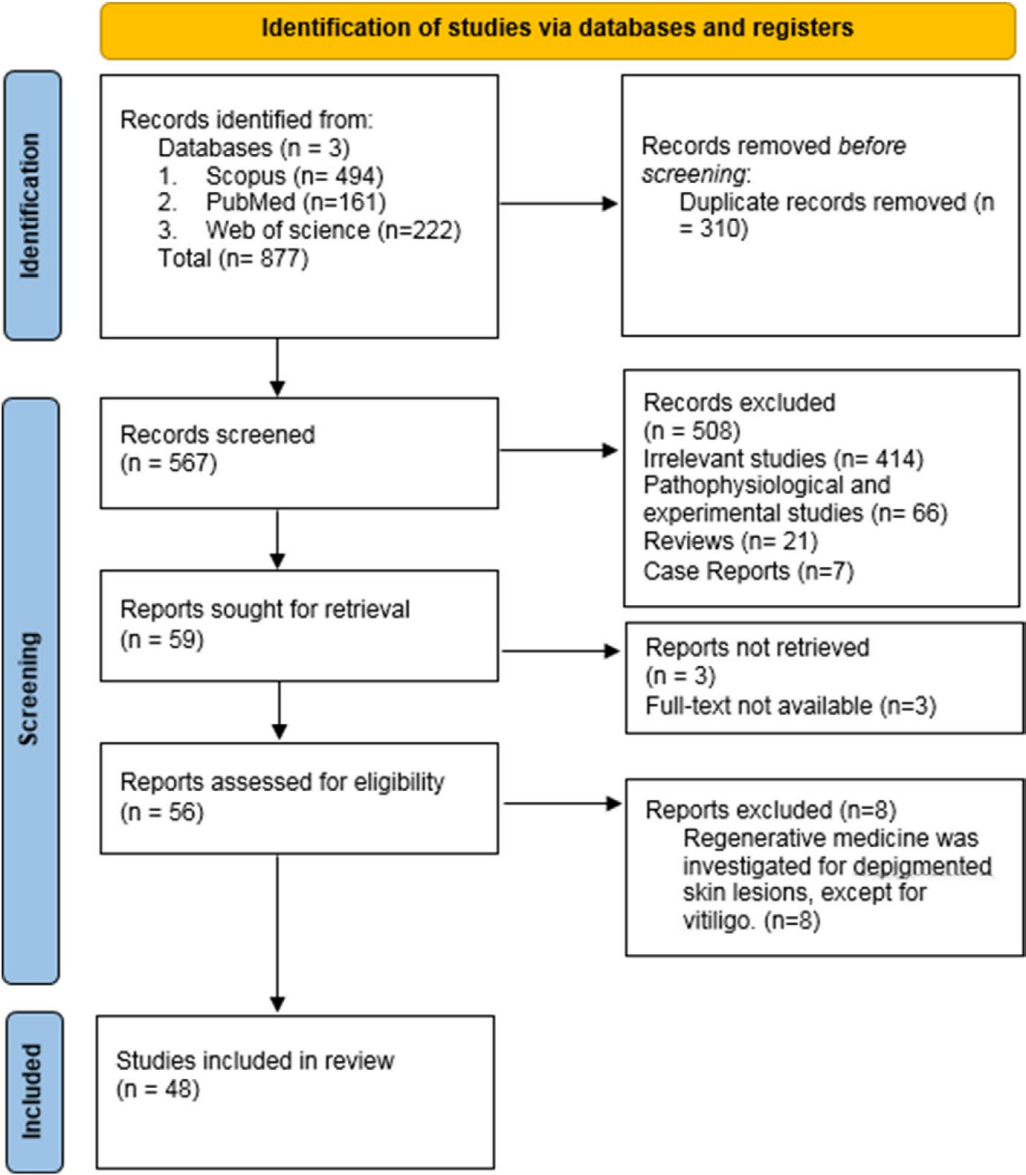
PRISMA statement of studied article.

In our initial search, 877 articles were obtained, and 48 articles were selected based on the inclusion and exclusion criteria to extract the final data. The screening steps are shown in the PRISMA chart (Figure 1). Additionally, the data extracted from the selected studies are presented in Table 2. In a total of 48 studies, 2186 patients with vitiligo were examined, out of which 1056 were women (48.3%) and 772 were men (35.3%). Furthermore, in 10 studies (16.4%), the separation of gender was not conducted.

**TABLE 2:** A summary of reviewed literature.

Author, Year	Design of study	Study sample	Age (Mean)	Sex ratio (F: M)	Fitzpatrick skin types	Previous treatment	Intervention method	Effectiveness	Adverse events
Esmat et al. [12], 2020	Randomized clinical trial	18 patients are randomly divided into two groups of 9 people.	20	14:4	3	Tofacitinib tablet	Group A utilized Ham F12 medium as a solution for MKTP, whereas Group B was provided with a blend of 5 ng/mL recombinant basic fibroblast growth factor and 5 mg/100 mL cyclic adenosine monophosphate integrated into the environment employed for MKTP. Both groups underwent NB‐UVB treatment twice weekly over a span of 24 weeks	After receiving MKTP, both groups saw a significant decrease in the size of their vitiligo lesions. However, group B participants experienced a greater reduction in area compared to group A participants (70% in group A versus 90% in group B)	Infection at donor site
Elgarhy et al. [10], 2020	Randomized clinical trial	40 patients with stable vitiligo	31.20 ± 12.68	28:12	4	Tacrolimus ointment and topical corticosteroid	For each patient, one or more patches were treated with autologous non‐cultured, non‐trypsinized melanocyte‐keratinocyte graft homogenized in plasma gel, followed by 16 sessions of NB‐UVB therapy. Furthermore, within the same anatomical site, one or more patches were solely treated with NB‐UVB as a control group	The improvement percentage in the treated patches, varied between 23.33% and 100%. The average improvement was 74.50%, 26.19%, and the median improvement was 80.83%	Pain
Shahbazi et al. [17]	Single‐arm clinical trial	38 patients with stable vitiligo	28.8 ± 11	22:16	3	Topical corticosteroid, and tofacitinib tablet	All patients underwent autologous melanocyte/keratinocyte transplantation as an intervention	After 6 months of follow‐up, the treated patients had varying responses to the therapy. Approximately 12.8% of them showed an excellent response, 36% of patients exhibited a good response, and the majority, accounting for 51.2%, had a moderate to minimal response to the treatment	None
Elazim et al. [3], 2020	Randomized clinical trial	53 patients with 94 lesions were categorized into three distinct groups	24.19 8.54	42:11	3	Tacrolimus ointment and topical corticosteroid	In the study, three groups were observed: Group 1 had 16 participants with 30 lesions who underwent FUE alone. Group 2 consisted of 18 participants with 32 lesions who received FUE in combination with topical CBD. Group 3 included 19 participants with 32 lesions who underwent FUE along with NB‐UVB phototherapy	In the second week, both Group 2 and Group 3 showed the quickest repigmentation onset, with rates of 16.7% and 10.5%, respectively. Overall, Group 2 demonstrated the most favorable response according to all evaluation methods	Folliculitis, inclusion cyst, Cobblestone appearance
Abdelghani et al. [1], 2017	Prospective, randomized comparative trial	Eighty patients participated in this study	33.83	50:30	3	Tacrolimus ointment and topical corticosteroid	The treatment options used in this study included fractional CO_2_ laser, PRP, a combination of fractional CO_2_ laser and PRP, and a combination of fractional CO_2_ laser and NB‐UVB therapy. The duration of the treatment period was 2 months. Clinical assessments of the patients were conducted 3 months after the completion of the final treatment	The group that received a combination of laser and PRP treatment showed the most favorable outcomes in terms of both repigmentation and patient satisfaction. In the PRP group, 20% of patients experienced repigmentation of more than 75%	None
Afify et al. [2], 2021	Randomized clinical trial	This study involved 20 patients who had a minimum of 6 patches of vitiligo	46.5 ± 14.5	11:9	4	Tofacitinib tablet	Each treatment group was assigned randomly to receive one of the following: PRP alone, fractional CO_2_ laser alone, combined fractional CO_2_ with NB‐UVB, combined fractional CO_2_ with PRP, combined fractional CO_2_ with PRP and NB‐UVB, or left untreated as a control group	There was a statistically significant improvement in CO_2_ laser with PRP group, and CO_2_ laser With PRP and NB‐UVB group	Erythema
Gupta et al. [31]	Randomized comparative trial	32 patients with vitiligo were divided into two groups: one comprising 15 individuals and the other consisting of 17 individuals	21.03	24:8	Unknown	Tacrolimus ointment and topical corticosteroid	Patients receiving the melanocyte‐keratinocyte transplantation procedure underwent different methods for preparing the recipient site. Group A used an Er:YAG laser, while Group B used a motorized dermabrader. The patients from both groups were evaluated for repigmentation levels after 1, 3, and 6 months	The comparison between the two groups did not reveal any significant statistical difference in terms of the total repigmentation achieved (54.67% for Group A and 48.841% for Group B, *p* = 0.663)	Hyperpigmentation, achromatic fissures, scarring and Re‐activation of disease
Elsaadany et al. [36], 2021	Randomized, comparative study	The study involved 36 patients who had approximately the same number of symmetrical patches of stable vitiligo, totaling 72 patches	Unknown	Unknown	3	Tacrolimus ointment and topical corticosteroid	Each patch was randomly assigned to one of two treatment groups. In group A, the patches received MEL therapy twice a week along with bi‐weekly intradermal PRP. In group B, the patches received MEL therapy alone. The treatment duration was a maximum of 4 months or until complete repigmentation occur	No statistically significant difference was observed between the two groups	Unknown
Deng et al. [37], 2020	Randomized clinical trial	The study consisted of 60 individuals diagnosed with stable vitiligo	40.35	34:26	3	Topical corticosteroid	Patients were randomly assigned to three different treatment groups. Group I received intradermal PRP injection, Group II received 308‐nm excimer laser alone, and Group III received 308‐nm excimer laser in combination with PRP injection. All treatments were administered for a duration of 3 months	Group III exhibited the highest score, followed by group II, and then group I. In terms of repigmentation responses, there were also notable distinctions among the groups (*p* < 0.001), with group III demonstrating the most favorable outcome	None
Agarwal et al. [39], 2021	Prospective, open‐labeled, comparative study	The study was conducted on 20 patients	25.65 ± 10.09	15:5	Types 2 and 3	Topical corticosteroid	The standard treatment given to all patients was PUVASOL. As an additional treatment, PRP or PPP was injected into two different areas, while the third area served as a control and received only PUVASOL. The patients were monitored for 4 weeks after completing the four treatment sessions	The areas treated with a platelet preparation (PRP or PPP) along with PUVASOL showed a significant increase in repigmentation. However, there was no significant difference observed between the PRP and PPP group (*p* = 0.824)	Mild pain and redness
Dev et al. [16], 2021	Single‐arm clinical trial	26 individuals with vitiligo and a total of 52 patches, which had been stable for at least 1 year, were selected as participants for the study	23.15	17:9	Types 3 and 4	Tacrolimus ointment	The researchers conducted NCES on two patches for a group of 26 vitiligo patients. The degree of repigmentation was evaluated during follow‐up visits using both clinical examination and photographic assessment	Excellent repigmentation was seen in 29 out of 52 patches	Unknown
Zhou et al. [47], 2013	Single‐arm clinical trial	He study comprised a group of 23 individuals diagnosed with vitiligo	30.77	11:12	3	Oral corticosteroid mini pulse and pimecrolimus ointment	The suspension of melanocytes was spread over the area where the laser had removed the skin, with an average of 600–1000 cells per square millimeter. The re‐coloring of the treated area was then assessed at intervals of 10 days, 1 month, 2 months, 3 months, and 6 months after the transplantation	Out of the total patients, 52.17% (12 patients) experienced an excellent response. A good response was observed in 26.09% (6 patients)	Unknown
Zhang et al. [48], 2014	Randomized clinical trial	The study included 473 participants who had various forms of vitiligo	22.5	244:229	4	Unknown	Patients who received cultured autologous melanocyte transplantation were randomly assigned to one of four study groups. In Group 1, patients underwent 20 sessions of NB‐UVB treatment before the transplantation. In Group 2, patients received 30 sessions of NB‐UVB treatment after the transplantation. Group 3 experienced 20 sessions of NB‐UVB treatment before the transplantation and an additional 30 sessions afterward. Lastly, Group 4 solely underwent transplantation without any supplementary treatment	Group 3 had the highest positive response rate. When comparing the four groups, statistical analysis revealed a significant difference between them (*p* < 0.001)	None
Vinay et al. [42], 2015	Open label prospective clinical study	This study involved the inclusion of 30 patients diagnosed with vitiligo, and a total of 60 lesions were observed	21.10 ± 5.64	9:21	3	Tacrolimus ointment and topical corticosteroid	All individuals participating in the research were subjected to the NCORSHFS method for cellular grafting. The patients underwent follow‐up visits at the clinic on the 8th day and at 4, 8, 16, and 24 weeks after the transplantation procedure	Out of the 60 lesions observed, 21 of them (35%) showed an optimum response. Among these 21 lesions, ten of them have achieved excellent repigmentation	None
Ebadi et al. [4], 2015	Non‐randomized clinical trial	Ten patients with 39 vitiligo patches participated in this study	31.8	6:4	Types 2 and 3	Pimecrolimus ointment and topical corticosteroid	Nine patches were subjected to treatment using MKT alone, while an additional 10 patches underwent treatment with both MKT and an excimer laser. Furthermore, 10 patches were exclusively treated with the excimer laser, and a final 10 patches were left untreated as control patches	In the group of nine patches treated with non‐cultured MKT alone, there was a median percentage reduction of 15.9% in the depigmented area compared to the baseline. Significantly greater reductions in the depigmented area were observed in patches treated with the combination therapy of excimer and non‐cultured MKT, when compared to the other therapies and the untreated patches	Unknown
Vashisht et al. [44], 2020	Randomized, double‐blind, comparative, therapeutic trial	This study included 22 patients with stable vitiligo	Unknown	Unknown	Types 3 and 4	Tacrolimus ointment and topical corticosteroid	Three patches with similar characteristics were randomly assigned to three treatment groups for hair follicle cell suspension: (A) trypsin combined with collagenase, (B) trypsin alone, and (C) dermabrasion using only a vehicle. The process of repigmentation was evaluated throughout a 6‐month follow‐up period	The percentages of repigmentation observed in the different groups were as follows: group A (33.22%), group B (24.31%), and group C (16.59)	Unknown
Abdel halim et al. [25], 2023	Randomized clinical trial	The study included 15 patients with a total of 30 lesions	Unknown	Unknown	Types 2 and 3	Tacrolimus ointment and topical corticosteroid	Patients in the study underwent two different treatments for their lesions. One lesion was treated with NCECS suspended in PRP, while another comparable lesion was treated with NCECS in Ringer's lactate. Following the treatment, patients received excimer sessions three times per week for 3 months. After 8 weeks, a preliminary assessment of the patients was conducted	Both lesions treated with NCECS suspended in PRP and NCECS in Ringer's lactate showed a significant improvement in terms of the size and color of the affected areas. Importantly, there was no statistically significant difference in the results between the two treatments	Unknown
Toossi et al. [18], 2011	Randomized clinical trial	The study included eight patients who had a combined total of 14 vitiligo patches	Unknown	6:2	3	Tofacitinib tablet	The study compared the repigmentation outcomes of eight vitiligo patches treated with non‐cultured MKT and six patches treated with dermabrasion alone. The evaluation of repigmentation was done approximately 4 months after the transplantation procedure	Out of the eight lesions treated with non‐cultured MKT, four lesions exhibited 96%–100% repigmentation, and one lesion showed 65%–95% repigmentation	Mild pain
Nilforoushzadeh et al. [27], 2022	Single‐arm clinical trial	Fifteen patients were included in this study	35.9	8:7	4	Tacrolimus ointment and topical corticosteroid	In this study, a suspension of melanocyte‐keratinocyte cells was applied through spraying. Following the application of the cells, patients underwent microneedling treatment. Biometric examinations were conducted before and after transplantation	The color disparity between the affected area and the normal skin significantly decreased at one, two, and 6 months after treatment with the cell suspension. The reduction was measured at 48.95% compared to the pre‐treatment condition	Erythema at the treatment areas, scar in the donor location
El Hawary et al. [32], 2020	Non‐randomized clinical trial	A total of 12 cases of vitiligo were included in the study	33.4	6:6	4	Tacrolimus ointment and topical corticosteroid	Three different approaches were employed to treat vitiligo lesions on the fingers: minipunch grafting, melanocytes keratinocyte transplantation procedure (MKTP) with prior cryoblebbing, and full CO_2_ laser resurfacing of the recipient site	Among the total of 18 lesions that underwent cryoblebbing followed by MKTP, 4 of them exhibited repigmentation exceeding 75%. In contrast, in the group where laser resurfacing + MKTP was used, only 1 out of 17 lesions showed a repigmentation rate of 30%, and in the minipunch grafting group, 1 out of 17 lesions showed a repigmentation rate of 10%	None
El‐zawahry et al. [14], 2011	Single‐arm clinical trial	A total of 22 cases of vitiligo were included in the study	20.5	17:5	3	Tacrolimus ointment	The patients were subjected to treatment involving the transplantation of autologous melanocyte‐keratinocyte suspensions. Following a period of 6 to 17 months, the patients' response to the treatment was assessed based on the level of pigmentation observed	Out of the 22 cases of vitiligo included in the study, five patients (23%) demonstrated an excellent response, and seven patients (32%) showed a good response	Unknown
Gabr et al. [52], 2015	Randomized clinical trial	50 vitiligo patients were included in the study	Unknown	Unknown	3	Tacrolimus ointment, and topical corticosteroid	The participants were divided into two groups: Group I, consisting of stable vitiligo cases, and Group II, consisting of unstable vitiligo cases. The patients received the following treatments: Group IA: cellular suspension containing melanocytes, hyaluronic acid, and MSC. Group IB: placebo treatment using a melanocyte medium and hyaluronic acid. Group IIA: local treatment combined with intravenous MScs. Group IIB: local treatment combined with intravenous normal saline as a placebo	The treated groups showed a significant increase in the area of repigmentation compared to the placebo groups at 12, 24, and 48 weeks. By the end of the 48‐weeks period, 85% of patients in group IA demonstrated repigmentation	Unknown
Garg et al. [15], 2019	Single‐arm clinical trial	Ten vitiligo patients were included in the study	Unknown	2:8	4	Oral corticosteroid	The patients with vitiligo underwent a procedure in which autologous non‐cultured tiny fragments of the epidermis were combined with PRP and then transplanted onto vitiligo lesions that had been superficially ablated using a pixel erbium YAG laser	After the treatment, repigmentation was noticeable after just 2 weeks. Out of the 20 lesions observed, 12 (60%) exhibited an excellent response. Among these 12 lesions, 10 (50%) achieved complete repigmentation in 8 weeks	Mottled pigmentation
Ibrahim et al. [35], 2016	Non‐randomized clinical trial	A total of 60 patients were included in the Study	28 ± 5.65	34:26	4	Tacrolimus ointment, and topical corticosteroid	Each patient underwent two different treatments: on the left side of their body, they were treated with NB‐UVB alone, while on the right side, they received NB‐UVB therapy along with intradermal injection of PRP. This combined treatment was administered every 2 weeks for a duration of 4 months	The combination group (PRP plus NB‐UVB) demonstrated a significant and positive improvement in repigmentation compared to the NB‐UVB group	Pain during injection and ecchymosis
El‐mongy et al. [11], 2023	Randomized clinical trial	A total of 20 patients were included in the Study	Unknown	Unknown	Types 2 and 3	Topical corticosteroid	Each patient will undergo two different treatments for their body: PRP on one side and a combination of Fractional CO_2_ laser and PRP on the other side. They will receive a total of six treatment sessions, with a two‐week gap between each session, over a span of 2.5 months. After the last session, there will be a follow‐up period of 3 months to monitor the results	According to the study, the combination of laser and PRP treatment resulted in superior outcomes compared to PRP treatment alone in terms of patient satisfaction and improvement. The frequency of excellent improvement was higher with laser and PRP treatment, whereas lower grades of improvement (no improvement, minimal improvement, and moderate improvement) were more common with PRP treatment alone	Unknown
Khattab et al. [38], 2020	Randomized clinical trial	A total of 52 patients were included in the Study	25.16	44:8	Unknown	Tacrolimus ointment	Group I, consisting of 26 patients, received treatment with intradermal PRP injection combined with the excimer laser. On the other hand, group II, also consisting of 26 patients, received treatment with only the excimer laser. In group I, the PRP injection was administered every 3 weeks for a total of 4 months, while the excimer laser treatment was performed twice a week for 16 weeks until a complete response was achieved	Group I exhibited a notably higher treatment response rate compared to group II, and this difference was statistically significant. Additionally, there was a significant disparity in Visual Analog Scale (VAS) scores between the two groups (*p* < 0.000)	Pain and erythema
Tawfik et al. [28], 2019	Non‐randomized clinical trial	A total of 42 patients were included in the Study	23.57	29:13	3	Tacrolimus ointment and topical corticosteroid	The patients were divided into two groups: Group I, consisting of 21 patients with 50 lesions treated using MKTP with a D/R ratio of 1/3 (3000 ± 500 cell/mm^2^), and Group II, comprising 21 patients with 52 lesions treated using MKTP with a D/R ratio of 1/10 (1000 ± 200 cell/mm^2^). Each patient's lesions were further subdivided into subgroups (a, and b) based on whether they received adjuvant phototherapy NB‐UVB. Subgroups Ia and IIa did not receive NB‐UVB, while subgroups Ib and IIb received adjuvant NB‐UVB	The average percentage of repigmentation was significantly higher in Group I compared to Group II, regardless of whether adjuvant NB‐UVB was administered. In subgroups Ia and Ib, the mean percentage of repigmentation was 86.00% ± 16.21% and 87.62% ± 11.66%, respectively. In contrast, in subgroups IIa and IIb, the mean percentage of repigmentation was 24.14% ± 18.08% and 29.98% ± 16.34%, respectively	Unknown
Singh et al. [29]	Randomized clinical trial	A total of 30 patients with 47 vitiligo lesions were included in the Study	22	20:10	4	Tacrolimus ointment and topical corticosteroid	Patients in Group 1 received treatment with NCES, whereas patients in Group 2 were treated with NCORSHFS. After a 16‐week period following surgery, the patients were assessed for the degree of repigmentation, color matching, changes in Dermatology Life Quality Index (DLQI) score, and overall patient satisfaction	In the NCES group, 83% of lesions showed excellent repigmentation, while in the NCORSHFS group, 65% of lesions demonstrated the same level of repigmentation (*p* = 0.154). Furthermore, the NCES group demonstrated good to excellent repigmentation in 92% of lesions, while the NCORSHFS group showed similar results in 78% of lesions (*p* = 0.425)	Mild pain and erosion
Mohamed et al. [49], 2023	Randomized clinical trial	A total of 40 patients with 40 vitiligo lesions were included in the Study	Unknown	Unknown	Types 2 and 3	Topical corticosteroid	The study focused on patients who underwent suspension transplants of melanocytes for treatment. The patients were divided into two groups: group A, where the recipient site was prepared through dermabrasion, and group B, where microneedling was used. After 3 months, the patients were assessed to measure the extent of repigmentation	Both methods, dermabrasion and microneedling, were found to be successful in achieving repigmentation in the patients. However, the group that underwent dermabrasion demonstrated a notable and statistically significant improvement	Unknown
Abalat et al. [50], 2022	Randomized clinical trial	A total of 40 patients were included in the Study	Unknown	Unknown	Types 3 and 4	Topical corticosteroid	The participants were divided into two equal groups for the study. Group A received treatment with RL‐suspended NCES, while group B received treatment with PRP‐suspended NCES. All patients were monitored for a period of 6 months to evaluate the effectiveness of the treatment in terms of clinical outcomes	Patients who underwent treatment with PRP‐suspended NCES demonstrated a notably greater repigmentation response in comparison to those who received RL‐suspended NCES. This significant difference was observed at 1, 3, and 6 months following the treatment	Unknown
Raizada et al. [34], 2021	Randomized clinical trial	A total of 58 patients were included in the Study	31.78	36:22	3	Tacrolimus ointment and topical corticosteroid	An equal number of patients were randomly assigned to two groups. Group A underwent a treatment regimen consisting of FCO_2_ laser combined with intralesional PRP, while Group B received FCO_2_ laser treatment alone. Each group received a single therapy session and were monitored on a monthly basis for a total duration of 3 months	The Group A showed a significantly greater reduction in VASI score compared to Group B throughout the follow‐up visits. In Group A, the mean ± standard deviation (SD) reduction in VASI score was 9.5 ± 0.22, 5.8 ± 1.12, and 3.6 ± 1.81, while in Group B it was 11.9 ± 2.83, 9.9 ± 3.11, and 8.9 ± 3.46 at each respective follow‐up visit	Erythema
Kumar et al. [45], 2018	Single‐ arm clinical trial	A total of 25 patients were included in the Study	24.5 ± 3.06	15:10	4	Tacrolimus ointment, topical corticosteroid, and tofacitinib tablet	Fifty hair follicles were taken from the back of the scalp and treated with a combination of trypsin and ethylenediaminetetraacetic acid (EDTA) to isolate the outer root sheath cells. The resulting cell mixture was filtered and centrifuged to collect a concentrated cell pellet. This pellet was then suspended again and applied to the recipient area that had undergone dermabrasion	After 6 months, the average (±SD) repigmentation observed was 52% ± 25.1%. Furthermore, among the total 25 patients, 8 of them (32%) achieved more than 75% repigmentation	Recipient site infection and color mismatch
Mastuzaki et al. [54], 2013	Single‐ arm clinical trial	A total of 27 patients were included in the Study	24.7	7:20	3	Tacrolimus ointment	The patients were subjected to treatment using keratinocytes derived from primary culture through Green's techniques or obtained from the first passage. To detach the stratified cultured epithelial sheets from their culture dishes, Dispase treatment was employed	Out of the patients diagnosed with segmental vitiligo, 12 individuals achieved a good therapeutic outcome following their initial surgery. This number increased to 14 when patients who underwent multiple surgeries were taken into account. There were six patients who experienced fair outcomes while no patients had poor outcomes	None
Challa et al. [30], 2022	Randomized clinical trial	A total of 74 patients were included in the Study	Unknown	Unknown	3	Tacrolimus ointment, and topical corticosteroid	Participants were divided into two groups through a random selection process: one group received ECS and the other group received HFCS. Both types of cell suspensions were examined to determine the overall number of cells, cell viability, and melanocyte count. The extent of repigmentation was evaluated at regular intervals over a period of 36 weeks	The percentage of repigmentation was significantly higher with ECS compared to HFCS at week 4 (*p* = 0.01) and week 16 (*p* = 0.03). However, there was no significant difference observed at week 24 (*p* = 0.38) and week 36 (*p* = 0.05)	Unknown
Shah et al. [41], 2016	Single‐ arm clinical trial	A total of 25 patients were included in the Study	Unknown	Unknown	4	Baricitinib tablet	The follicular units were obtained using the Follicular Unit Extraction method. The outer root sheath cells were separated through trypsinization. Afterwards, the solution containing these cells was transplanted onto a recipient site that had undergone dermabrasion	The average repigmentation rate was 80.15% with a standard deviation of 22.9%. In 60% of the patients, the repigmentation was deemed excellent, ranging from 90% to 100%	Unknown
Nilforoushzadeh et al. [19], 2022	Single‐arm clinical trial	A total of 15 patients were included in the Study	Unknown	Unknown	3	Tacrolimus ointment	After performing dermabrasion, a cell suspension containing melanocytes and keratinocytes obtained from the patients themselves was transplanted to the respective area. The participants in this study underwent assessments using VisioFace, MPA9, and Skin Scanner‐DUB at four different time points: before transplantation, and then at 1, 2, and 6 months post‐transplant	The difference in color between the affected area and the normal skin showed a notable decrease after 1, 2, and 6 months of melanocyte transplantation in comparison to before the procedure (13.8 ± 0.45 initially, and 12.9 ± 0.43, 12.2 ± 0.45, and 10.2 ± 0.34 at months 1, 2, and 6 respectively, *p* < 0.001)	Unknown
Donaparthi et al. [46], 2016	Non‐Randomized clinical trial	A total of 11 patients were included in the Study	24.09	6:5	Types 3 and 4	Oral corticosteroid mini pulse and pimecrolimus ointment	The sites were divided into two equal groups, each containing 30 sites. One group was treated with epidermal melanocyte transfer (EMT), while the other group received hair follicular melanocyte transfer (HFMT). The success of the treatments was assessed by measuring the extent of repigmentation. The final evaluation, measuring the affected area, was conducted 6 months after the completion of the treatment	After 6 months, the EMT group showed a repigmentation rate exceeding 90% in 83.33% of their patches, while the HFMT group had a rate of 43.33%. In terms of repigmentation exceeding 75%, Group A had a rate of 90% in their patches, whereas Group B had 43.34%. There was a statistically significant difference in overall pigmentation between the two groups	Perigraft halo, color mismatch and invisible scarring.
Rekik et al. [59], 2022	Single‐arm clinical trial	A total of 10 patients were included in the Study	36.2	2:8	Types 3 and 4	Tacrolimus ointment, topical corticosteroid, and tofacitinib tablet	Every patient in our study received platelet‐rich plasma. On average, our patients underwent 2.6 sessions of platelet‐rich plasma treatment, with a range of 1 to 6 sessions	After approximately 1.5 sessions, there was a noticeable improvement in the appearance of the skin lesions. About 40% of patients experienced more than a 50% repigmentation in at least one lesion. In two cases, a significant improvement of over 75% (grade 4) was observed after an average of 5.5 sessions. Additionally, 40% of patients achieved more than a 50% repigmentation in at least one lesion	Unknown
Thakur et al. [43], 2015	Single‐arm clinical trial	A total of 50 patients were included in the Study	23.7	20:30	3	Tacrolimus ointment, and topical corticosteroid	Fifty individuals with stable vitiligo who had 63 patches of discoloration in non‐hair‐bearing areas underwent treatment with follicular unit grafts. The researchers assessed the decrease in the size of vitiligo patches and the improvement in the accompanying white hair through both subjective and objective evaluations	Out of the total 63 patches, good to excellent response was seen in 39 (61.9%) lesions. The mean improvement seen was 61.17%	Intraoperative bleeding at the recipient site and inclusion cyst
Parambath et al. [26], 2019	Randomized clinical trial	A total of 21 patients were included in the Study	Unknown	Unknown	4	Tofacitinib tablet	The two patches affected by vitiligo were assigned randomly to receive either NCES suspended in PRP or PBS (phosphate‐buffered saline). Following the surgery, patients underwent heliotherapy for 15 min each day, starting from 1 week after the operation	After a period of 6 months, the average repigmentation achieved through the area method was 75.6% ± 30% SD in the PRP arm, while in the non‐PRP arm, it was 65% ± 34% SD. This difference was statistically significant, with a *p*‐value of 0.0036. Additionally, patient satisfaction, as measured by a visual analogue scale, was higher in the PRP arm compared to the non‐PRP arm at the 6‐month mark, with a *p*‐value of 0.001	Unknown
Mrigpuri et al. [20], 2019	Randomized clinical trial	A total of 30 patients were included in the Study	24.23 ± 5.81	16:14	Unknown	Unknown	The patients in the study were randomly assigned to two groups: one group receiving NCES prepared using the 4C method and the other group receiving NCES prepared in the laboratory (lab‐NCES). A blinded observer evaluated each patient at 4, 8, and 16 weeks after surgery. The evaluations focused on assessing the extent of repigmentation, color match, patient global assessment (PGA), and pattern of repigmentation	The comparison between the 4C method and lab‐NCES in terms of repigmentation outcomes showed similar results. The percentage of excellent repigmentation (≥ 90%) was 34% for the 4C method and 37% for lab‐NCES, with no significant difference (*p* = 1.000)	Surgical site infection
Nareswari et al. [51], 2019	Case series	A total of 7 patients were included in the Study	33.4	5:2	Types 3 and 4	Tacrolimus ointment, and topical corticosteroid	Autologous non‐cultured epidermal cell suspension (NCECS) in combination with PRF transplantation was used to treat seven cases of stable vitiligo. The researchers captured clinical photographs before the procedure, as well as at week 1, 4, and 16, in order to assess the pigmentation changes and color match. The designated endpoint for evaluating the results was 24 weeks after the transplantation procedure	The majority of patients in the study experienced the onset of repigmentation between 4 and 24 weeks following their surgery. This repigmentation was characterized by the emergence of normal pigmented macules and/or patches	Unknown
Orouji et al. [24]	Single‐arm clinical trial	A total of 300 patients were included in the Study	27.1 ± 9.7	189:111	Unknown	Unknown	The researcher prepared the epidermal cell suspension by processing the patient's own skin sample. This cell suspension was then injected into a total of 1060 vitiligo patches across 300 patients. Throughout the study, patients did not receive any additional phototherapy. At regular follow‐up visits, both an experienced dermatologist and the patients themselves assessed and recorded the repigmentation score and self‐assessment score for up to 30 months after the treatment	The average repigmentation score 3 months after transplantation was 1.12 ± 0.73. Notably, there was a significant increase in the average repigmentation score until 9 months after cell transplantation, reaching 1.98 ± 1.20. At the nine‐month mark, more than 50% repigmentation was observed in 32.2% of the treated areas	Mild pain at the donor site, keloid Formation at the donor site and Post‐inflammatory hyperpigmentation
Shahbazi et al. [23], 2020	Single‐arm clinical trial	A total of 39 patients were included in the Study	34.33	15:24	Types 3 and 4	Tacrolimus ointment	Partial grafts were obtained from the gluteal areas of all patients. Epidermal cells, including non‐cultured melanocytes and keratinocytes, were isolated using an enzyme‐based method and found to have a viability of more than 98%. These cells were then injected into the epidermal layer of the skin. After a one‐year period of monitoring, noticeable repigmentation was observed	The results obtained validated that the mixture of epidermal cells in the cellular suspension effectively enhanced the rate of repigmentation, leading to the restoration of the normal color	None
Khalil et al. [33], 2023	Randomized clinical trial	A total of 30 patients were included in the Study	23.73 ± 3.83	12:18	Types 3 and 4	Tacrolimus ointment and Baricitinib tablet	Each participant had three lesions, and they were randomly divided into two groups: one group received PRP injections, and the other group underwent microneedling. Both groups received their respective treatments every 2 weeks for a total of four sessions. The third lesion served as a control and received no treatment. Throughout the study, all lesions, including the control, were exposed to NB‐UVB three times a week	The results indicate that PRP injection showed better outcomes compared to microneedling in terms of repigmentation, complications, and patient satisfaction. The average rates of repigmentation in the lesions treated with PRP, microneedling, and the control group were 58.17 ± 21.52, 24.5 ± 18.77, and 15.17 ± 13.49, respectively (*p* < 0.001)	Unknown
Ramos et al. [22], 2017	Case series	A total of 20 patients were included in the Study	30.75 ± 12.2	14:6	3	Tacrolimus ointment	In the duration of the research, a group of 20 patients underwent a total of 24 procedures involving the transplantation of their own skin cells (melanocytes and keratinocytes) to treat vitiligo in various areas of the body. The outcomes were assessed within a timeframe of 3–6 months following the interventions	Out of the 20 patients who received a total of 24 procedures, 25% exhibited an outstanding level of repigmentation, 50% experienced satisfactory repigmentation, 15% had moderate repigmentation, and 10% showed a poor response	Pain, burning sensation, and Hyperchromia in the receptor area
Razmi et al. [21], 2018	Randomized Clinical Trial	A total of 30 patients were included in the Study	23.4	18:12	4	Tacrolimus ointment, topical corticosteroid, and tofacitinib tablet	The ECS + FCS mixture was created by combining equal amounts of FCS and ECS based on cell numbers. Following manual dermabrasion, ECS was applied to one lesion, while ECS + FCS was applied to the corresponding anatomically based lesion of the same patient. A blinded observer conducted follow‐up evaluations at 4, 8, and 16 weeks, recording the extent of repigmentation, color match, pattern of repigmentation, patient satisfaction, and any complications that arose	The combination of ECS and FCS yielded better results in terms of repigmentation compared to ECS alone. The results showed that the combined treatment had better outcomes in terms of the extent of repigmentation compared to the alternative treatment (76% vs. 57%, *p* < 0.001)	Hyperpigmentation at the skin donor
Salem et al. [40], 2021	Randomized clinical trial	A total of 17 patients were included in the Study	24.7 ± 10.32	8:9	Types 2 and 3	Baricitinib tablet	In this study, a total of 34 vitiliginous patches, with two patches per patient with stable vitiligo, were included. The patches were treated with autologous MPG and exposed to phototherapy. Additionally, some patches received enhancement through the PRP procedure during the initial treatment and monthly for the following 3 months. The repigmentation progress was evaluated using vitiligo scores, and the levels of lesional bFGF were measured	At the conclusion of the 20‐week study, ideal repigmentation (> 75% repigmentation) was observed in 10 out of the total patches treated with the MPG/phototherapy combination and in 12 patches treated with the PRP‐assisted method. However, this difference was not found to be statistically significant (*p*‐value = 0.625)	Cobblestone appearances, pain, perigraft halo, infection, and static graft

Out of 48 studies, the average age of the participants was reported in 35 studies, with the average age of the patients being 29.7 years. Among these studies, 26 were randomized clinical trial studies (54.2%), 15 were single‐arm clinical trial studies (31.2%), 5 were non‐randomized clinical trial studies (10.4%), and 2 studies (4.2%) were case series.

Among the 48 studies conducted, there were a total of 96 intervention groups, out of which 76 were treated using regenerative medicine methods. The following information provides the number and percentage of utilization for each regenerative medicine method:

- Transplantation of autologous epidermal melanocyte/keratinocyte cells in 30 groups (39.5%).
- Platelet‐rich plasma injection (PRP) in 15 groups (19.7%).
- Cell transplantation with hair follicle origin in 11 groups (14.5%).
- Isolated melanocyte transplantation in 10 groups (13.2%)
- Melanocyte‐keratinocyte suspension in PRP in 4 groups (5.3%)
- Combined cell transplantation with epidermal and hair follicle origin in 1 group (1.3%)
- Combination of melanocyte‐keratinocyte transplantation and plasma‐rich fibrin (PRF) injection in 1 group (1.3%)
- Platelet‐poor plasma (PPP) injection in 1 group (1.3%)
- Systemic injection of mesenchymal stem cells in 1 group (1.3%)
- Cultured keratinocytes transplantation in 1 group (1.3%)
- A suspension consisting of melanocytes and mesenchymal stem cells in 1 group (1.3%)

In the following section, we will examine each regenerative medicine method. It is important to note that the total number of patients in each group might not match the number of patients mentioned earlier due to the selection of multiple modalities for several lesions in some patients. The recovery rate of vitiligo lesions treated with a combination of laser therapy, phototherapy, and regenerative medicine is shown in Tables 3 and 4.

**TABLE 3:** Effectiveness of the combination of laser and regenerative medicine in vitiligo treatment.

The combination of fractional CO_2_ laser and PRP	20% of patients experienced repigmentation of more than 75% The mean ± standard deviation (SD) reduction in VASI score was 9.5 ± 0.22
The combination of melanocyte‐keratinocyte transplantation and Er:YAG laser therapy	Total repigmentation achieved was 54.67%
The combination of fractional CO_2_ laser and of melanocyte‐keratinocyte transplantation	Lesions showed a repigmentation rate of 30%

**TABLE 4:** Effectiveness of the combination of phototherapy and regenerative medicine in vitiligo treatment.

Combination of MKTP and NB‐UVB	70% reduction in the depigmented area The median improvement reached 80.83%
Combination of fractional CO_2_ laser, PRP, and NB‐UVB	The total repigmentation obtained was 42.34%
Combination of PRP, and NB‐UVB	The average rate of repigmentation in the lesions was 58.17 ± 21.52
Combination of cultured autologous melanocyte transplantation and narrowband UVB	The mean repigmentation rate in the lesions was 43.17 ± 23.12

### 3.1 Transplantation of Autologous Epidermal Melanocyte/Keratinocyte

A total of 802 patients participated in 22 studies involving 30 intervention groups focused on epidermal melanocyte‐keratinocyte transplantation for vitiligo. Among these, 443 were female and 254 were male, with the average age being 26.21 years. Most studies assessed outcomes at a 6‐month interval, where significant repigmentation was observed, particularly in the study by Dev et al. [16], which reported over 90% repigmentation in 29 out of 52 patches (*p* ≤ 0.05). Overall, a good to excellent response was found in 48.8% of patients, with a 15.9% reduction in depigmented patches, while 62.5% experienced over 65% improvement. Within 9 months, 32.2% achieved more than 50% repigmentation.

Different cell suspension solutions demonstrated varied effectiveness, with Ringer's lactate and Ham F12 medium showing significant improvements. Basic fibroblast growth factor (bFGF) combined with cyclic adenosine monophosphate (cAMP) achieved a 90% reduction in depigmentation, while phosphate‐buffered saline (PBS) resulted in approximately 65% repigmentation [12, 25, 26, 27].

Combining melanocyte transplantation with NB‐UVB facilitated uniform repigmentation in 65% of patients, outperforming NB‐UVB alone [10]. The addition of excimer laser therapy led to a 41.9% reduction in depigmented patches, markedly more effective than transplantation alone (15.9% reduction).

Comparative studies indicated that epidermal origin cells yielded a 14% higher response rate than follicular origin cells after 4 months, although no significant differences were noted by the ninth month [29, 30]. Techniques such as microneedling following cell suspension spraying and cryoblebbing before transplantation showed promising repigmentation results, demonstrating an improvement above 75% in some patients [27, 32]. Overall, these findings underscore the effectiveness of regenerative treatments for vitiligo.

### 3.2 Platelet‐Rich Plasma Injection (PRP)

A total of 338 patients participated in 11 studies with 15 intervention groups focused on platelet‐rich plasma (PRP) treatment for vitiligo. Among them, 169 were women and 113 were men, with an average age of 25.55 years. Significant improvements were noted in 13 groups (*p* ≤ 0.05), while two groups showed no significant difference compared to controls. Khalil et al. [33] reported the highest average repigmentation rate of 58.7%.

Combining PRP with CO_2_ fractional laser resulted in over 75% repigmentation in 40% of patients after 3 months, which was significantly better than combinations with NB‐UVB or PRP alone [1]. Additionally, a combination of NB‐UVB, PRP, and CO_2_ laser achieved grade 2 repigmentation in 35% of patients, outperforming separate treatments [2]. The combination of excimer laser and PRP produced good to excellent responses in 80% of patients, significantly higher than either treatment alone [37, 38].

PRP combined with Psoralen + sunlight (PUVASOL) yielded excellent repigmentation in 40% of patients and a 63.6% reduction in depigmented patches compared to PUVASOL alone [39]. A study showed that PRP injection was superior to microneedling, achieving a repigmentation rate of 58.17% [33]. Furthermore, a comparison of PRP and punch grafting for vitiligo management indicated a repigmentation rate above 75% in 29.4% of punch grafting patients and 35.2% for PRP readers over 3 months [40]. Overall, these findings highlight the efficacy of PRP as a treatment modality for vitiligo.

### 3.3 Cell Transplantation With Hair Follicle Origin

A total of 268 patients participated in 9 studies involving 11 intervention groups focused on hair follicle‐derived cell transplantation for vitiligo. Among them, there were 102 women and 82 men, with an average age of 23.41 years. Notable improvements were observed in all studied groups (*p* ≤ 0.05), with Shah et al. [41] reporting a repigmentation rate of 80.15% ± 22.9%.

Cell transplantation from hair follicles yielded good to excellent repigmentation in 56.66% of patients within 4 months, increasing to 35% and 61.9% at 6 months [3, 42, 43]. Combining hair follicle transplantation with calcipotriol betamethasone dipropionate achieved good to excellent results in 93.8% of patients within 4 months, while the combination with NB‐UVB resulted in similar success for 81.2% of patients [3].

Techniques using trypsin and collagenase led to a repigmentation rate of 33.22%, while trypsin alone achieved 24.31%, both surpassing the control group's rate of 16.59% [44]. Using trypsin with EDTA resulted in a 52% ± 25.1% rate over 6 months, with over 75% repigmentation seen in 32% of patients [45].

Comparatively, epidermal cell transplantation showed a 92% rate of good to excellent repigmentation within 4 months, while follicular transplantation achieved 78%. In a 9‐month study, excellent repigmentation rates were 61.7% for epidermal and 53.2% for follicular cells, with no significant differences noted [29, 30, 42]. Furthermore, a separate investigation reported a 40% higher rate of excellent repigmentation for melanocyte transplantation from epidermal sources compared to follicular sources within 6 months.

### 3.4 Isolated Melanocyte Transplantation

A total of 572 patients participated in 5 studies across 10 intervention groups. Among them, 261 were women and 246 were men. In 2 studies, gender separation was not reported. The average age of the participants in the groups was 22.90 years, although the age of the patients was not clearly reported in 2 studies.

All ten groups investigating melanocyte transplantation for vitiligo treatment reported a significant improvement in repigmentation (*p* ≤ 0.05). Among the studies investigating the effectiveness of melanocyte transplantation in the treatment of vitiligo, Donaparthi et al.'s study [46] reported the highest repigmentation rate. The study revealed that for epidermal melanocyte transplantation, over 90% pigmentation was achieved in 83.33% of the patients within 6 months. As for follicular melanocytes, over 90% pigmentation was achieved in 43.33% of the patients.

In a similar study, a remarkable rate of good to excellent response was observed in 78.26% of patients within 6 months [47]. The utilization of 20 sessions of NB‐UVB prior to melanocyte transplantation, followed by 30 sessions afterwards, resulted in excellent repigmentation in 81% of patients. This figure represents a 37.4% increase compared to patients who solely underwent transplantation, and a 12.5% and 19.10% improvement compared to patients who received NB‐UVB only before or after transplantation [48].

Among the different methods of melanocyte transplantation, dermabrasion of the recipient area demonstrated significant enhancement in comparison to microneedling [49].

### 3.5 Melanocyte‐Keratinocyte Suspension in PRP

A total of 66 patients participated in four studies across four intervention groups. Among them, two were women, and eight were men. Gender separation was not reported in three of the studies. The average age of the participants was not clearly mentioned in any of the four studies.

Among the four studies that investigated the effectiveness of melanocyte‐keratinocyte suspension in platelet‐rich plasma (PRP), all four studies confirmed the significant effectiveness of this intervention by comparing repigmentation before and after treatment (*p* ≤ 0.05). Among the studies that investigated the effectiveness of keratinocyte‐melanocyte suspension in PRP, Garg et al.'s study [15] showed a complete repigmentation rate in 50% of patients within 8 weeks, while Parambath et al.'s study [26] reported an average repigmentation rate of 75.6% ± 30% within 6 months. Both studies demonstrated the highest rates of repigmentation.

In a comparison between cell suspension in PRP and cell suspension in Ringer's lactate solution, significant improvement was observed in both methods. However, no significant difference was found between the two methods within the 3‐month period. In a similar study, cell suspension in PRP resulted in 75.6% repigmentation, which was 10.6% higher than that of cell suspension in PBS (phosphate‐buffered saline) solution. Another study demonstrated significant improvement in the group receiving cell suspension in PRP compared to the group receiving cell suspension in Ringer's lactate solution over a 6‐month period [25, 26, 50].

### 3.6 Combined Cell Transplantation With Epidermal and Hair Follicle Origin

A total of 30 patients participated in a single study within one intervention group, comprising 18 women and 12 men. The average age of the participants was 23.4 years.

In one group that underwent intervention with a cell therapy of both follicular and epidermal origin, a significant difference was observed compared to the group receiving cells of only epidermal origin (*p* ≤ 0.05). Ramos et al. [22] reported a repigmentation rate of 76% over a 4‐month period for a combination of epidermal cell suspension and follicular cell suspension. This percentage was 19% higher than the group receiving epidermal cell transplant.

### 3.7 Combination of Melanocyte‐Keratinocyte Transplantation and Plasma‐Rich Fibrin (PRF) Injection

A total of 7 patients participated in a single study within one intervention group, comprising 5 women and 2 men. The average age of the participants was 33.4 years.

In the group receiving keratinocyte‐melanocyte transplantation in combination with plasma‐rich fibrin (PRF), a significant improvement in repigmentation was observed (*p* ≤ 0.05). The percentage of repigmentation among the subjects in this case‐series study varied from 5% to 94% [51].

### 3.8 Platelet‐Poor Plasma (PPP) Injection

A total of 20 patients participated in a single study within one intervention group, comprising 15 women and 5 men. The average age of the participants was 25.65 years.

The group receiving plasma‐poor platelet (PPP) treatment also showed a significant improvement in repigmentation, although there was no significant difference compared to the group receiving platelet‐rich plasma (PRP). The percentage of repigmentation in the PPP group was 25.61% higher than that of the control group. Specifically, the percentage of repigmentation was 58.2% in the PPP and PUVA group, compared to 32.59% in the PUVA‐only group [39].

### 3.9 Systemic Injection of Mesenchymal Stem Cells

A total of 25 patients participated in a single study within an intervention group, and the gender ratio and average age were not clearly defined. The group receiving systemic mesenchymal stem cells in combination with local treatments showed a significant improvement in the lesions, but this improvement did not differ significantly from the control group [52].

### 3.10 Cultured Keratinocytes Transplantation

A total of 27 patients participated in a single study within one intervention group, comprising 7 women and 20 men. The average age of the participants was 24.7 years. The study investigating the effectiveness of keratinocyte transplantation in the treatment of vitiligo reported that 12 out of 27 patients showed a repigmentation rate of over 90% [53].

### 3.11 A Suspension Consisting of Melanocytes and Mesenchymal Stem Cells

A total of 25 patients participated in a single study within an intervention group, and the gender ratio and average age were not clearly defined. When combining melanocyte transplantation with mesenchymal stem cell therapy, a notable repigmentation rate of 24.85% was achieved within 12 months. This represents an additional 11.50% improvement compared to melanocyte transplantation alone [52].

The results of our review have shown that other regenerative medicine methods, such as stromal vascular fraction, stem cell‐derived conditioned medium, and exosome, have not been clinically investigated for the treatment of vitiligo.

Side effects have been examined in 28 out of 48 studies, and they were observed in 21 of these studies. The most common complication reported was pain, which was mentioned in 9 studies. Additionally, hyperpigmentation at the site of tissue harvest was documented in 5 studies, erythema in 5 studies, infection in 4 studies, scar formation in 3 studies, inclusion cyst in 2 studies, cobblestone appearance in 2 studies, perigraft halo in 2 studies, color mismatch in 2 studies. Other complications such as bleeding, folliculitis, keloid formation, burning sensation, fissure, ecchymosis, and erosion were each reported in one study. Importantly, no serious or life‐threatening complications have been reported in any of the studies.

### 3.12 Side Effects and Contraindications

1. Platelet‐Rich Plasma (PRP): Adverse effects include injection site reactions such as pain, erythema, swelling, or bruising, as well as a slight risk of infection at the injection site and a transient increase in redness in the treated area immediately after the procedure [26, 33, 50].
  Contraindications for PRP include active infections in the area to be injected, coagulation disorders, where patients with bleeding disorders or those on anticoagulant therapy may face increased risks, and certain diseases including autoimmune disorders, skin infections, or any pathology affecting the treatment area [11, 34, 35, 38, 40].
2. Transplantation of Autologous Epidermal Melanocyte/Keratinocyte Cells (MKTP): Adverse effects include pain at the donor or recipient site, resulting in discomfort during and after the procedure, as well as a potential risk of infection at both the donor site and the transplanted area. There is also a possibility of hypertrophic scarring at the donor site, along with changes in skin pigmentation at the transplant site, which may manifest as hyperpigmentation or hypopigmentation [10, 12, 16, 30, 32].
  Contraindications include active skin diseases such as psoriasis or dermatitis in the area to be treated, severe chronic diseases that impair wound healing, and pregnancy or breastfeeding, for which caution is advised [25, 26, 27, 29].
3. Cell Transplantation With Hair Follicle Origin: Adverse effects include infection at graft or injection sites, posing a risk of contamination during the procedure, and folliculitis, which is the inflammation of hair follicles that can cause itchiness or pus formation. There may also be a poor graft take, where ineffective integration leads to inadequate results [3, 29, 42, 45].
  Contraindications include active infections in the transplantation area or underlying conditions affecting skin health, as well as an immunocompromised state, where patients unable to mount an adequate immune response face increased risks [30, 42, 43, 44].
4. Isolated Melanocyte Transplantation: Adverse effects include injection site reactions, such as swelling, pain, or bleeding at the transplant site, as well as transient erythema, which is redness following injections. There may also be a color mismatch, resulting in changes in pigmentation that do not blend seamlessly with the surrounding skin [46, 49].
  Contraindications include active dermatologic conditions like psoriasis or eczema, as well as scarring or dark spots at the treatment site due to prior trauma, which may interfere with results [47, 48].
5. Platelet‐Poor Plasma (PPP): Adverse effects may include mild pain at the injection site, localized bruising in the treated area, and transient itchiness or redness, particularly in sensitive patients [39].
  Contraindications for PPP include active skin infections or lesions, as the treatment should not be used in infected areas, patients with bleeding disorders due to a high risk for complications, and individuals with a history of adverse reactions to plasma products, as any known allergies to its components would be a concern [39].

### 3.13 Risk of Bias Assessment

We analyzed the risk of bias in clinical trials included in our review, assessing 43 out of 48 studies using the RoB 2 tool. The results for each domain are as follows:

- Randomization Process (D1): 44.19% of studies were rated as low risk, 9.30% had some concerns, 44.19% were high risk, and 2.33% provided no information.
- Deviations from Intended Interventions (D2): 37.21% were low risk, while 62.79% were high risk.
- Bias Due to Missing Outcome Data (D3): 62.79% were low risk, 37.21% had some concerns, and none rated as high risk.
- Measurement of the Outcome (D4): 95.35% were low risk, 4.65% had some concerns, and no studies were rated as high risk.
- Selection of the Reported Result (D5): All studies (100%) were rated as low risk, with no studies having some concerns or high risk.

**FIGURE 2:**
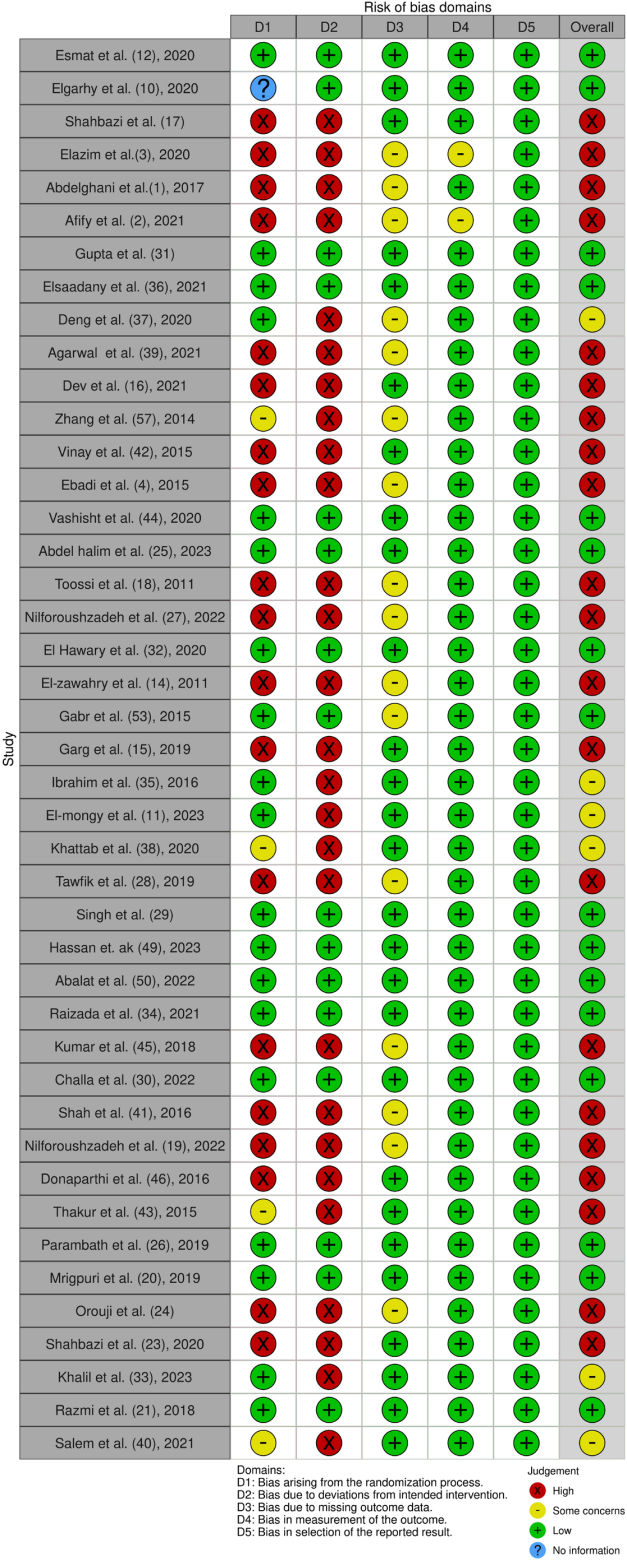
Summary of risk of bias across all domains for each included study.

**FIGURE 3:**
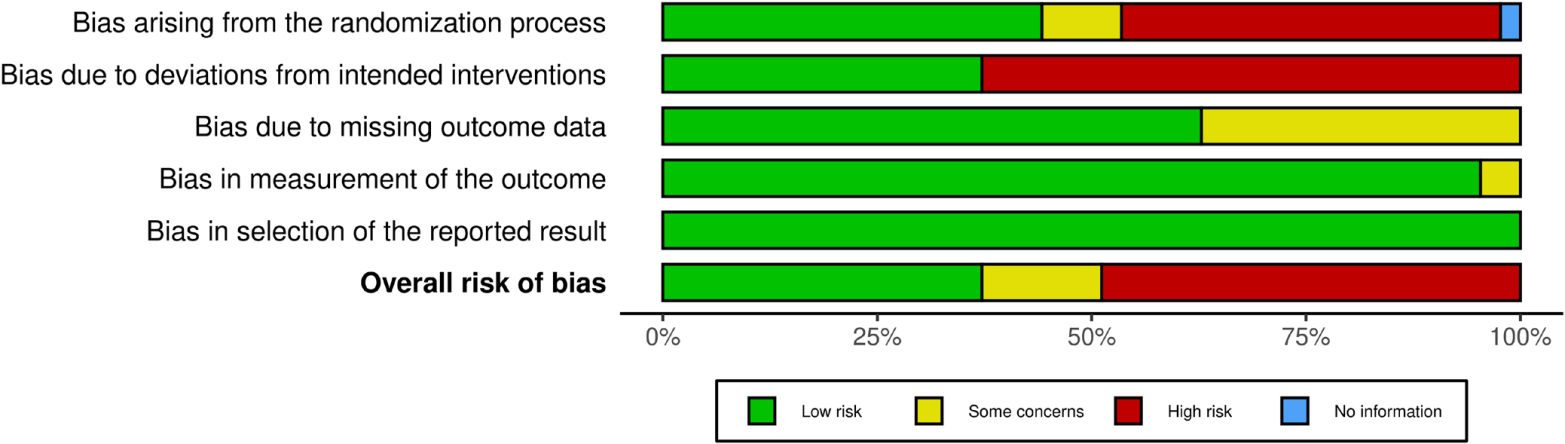
Overall risk of bias assessment for the included study.

Overall, 37.21% of studies were categorized as low risk, 13.95% had some concerns, and 48.84% were rated as high risk. To better illustrate these findings, we will enhance Figure 2 to include a detailed breakdown of the risk of bias across all domains for each included study. This will provide a clearer view of the risk levels and allow readers to grasp the nuances of the assessments conducted. Additionally, Figure 3 will continue to illustrate the overall risk of bias assessment for the studies.

## 4 Discussion

Regenerative medicine has found a special place in the treatment of skin diseases and injuries, with new applications being discovered daily [54]. This systematic review investigates the use of regenerative medicine methods in the treatment of vitiligo, revealing several effective therapeutic approaches. These methods include melanocyte‐epidermal keratinocyte transplantation, platelet‐rich plasma (PRP) injection, platelet‐poor plasma injection, cell therapy based on hair follicle transplantation, melanocyte transplantation, keratinocyte transplantation, melanocyte‐keratinocyte suspension in PRP, and systemic injection of mesenchymal stem cells.

Among these, epidermal melanocyte‐keratinocyte transplantation has been one of the most common methods for treating vitiligo since 1992. This technique involves harvesting tissue from a donor site (usually the gluteal region), preparing the melanocyte‐keratinocyte complex in vitro, and then suspending it in the recipient site after removing the epidermis and superficial dermis [4, 54]. Our study demonstrated the effectiveness of this technique, with all 30 intervention groups showing significant improvement. Moreover, studies utilizing PRP for processing these cells showed better results compared to control groups, as PRP enhances the release of basic fibroblast growth factor and other chemokines, promoting melanocyte migration and proliferation of keratinocyte and melanocyte cells. PRP can be used either as a suspension containing melanocyte‐keratinocyte cells or via intradermal injection [55, 56].

Cell therapy through hair follicle transplantation has also shown significant therapeutic success due to the presence of melanocytes in the outer root sheath of hair follicles [41, 46]. When inactive melanocytes are transplanted, they proliferate and migrate to the epidermis and hair matrix, promoting healing of vitiligo lesions. Importantly, the ratio of melanocytes to keratinocytes in hair follicles is 6–36 times higher than in the epidermis, underscoring the importance of this therapy [3, 45]. Singh et al. [29] found that non‐cultured extracted hair follicle outer root sheath cell suspension provided a better therapeutic response than autologous non‐cultured epidermal cell suspension for treating vitiligo.

Our review also shows that the use of cultured or non‐cultured melanocytes can lead to significant improvement in vitiligo treatment. The method of cell transplantation is similar to melanocyte‐keratinocyte transplantation but only involves placing melanocytes at the recipient site. Following melanocyte transplantation, the release of numerous cytokines occurs, inhibiting CD8+ T cell activity. This mechanism, alongside the activity of the melanocytes, contributes to repigmentation of the lesions [23, 47, 57]. Notably, Donaparthi et al. [46] found that melanocytes from the epidermis produced a higher percentage of repigmentation compared to follicular melanocytes. Challa et al. [30] confirmed that epidermal cell transplantation was superior to follicular cell transplantation in the first 4 weeks, although no significant difference was observed between the two methods at weeks 24 and 36.

The effectiveness of regenerative medicine in treating vitiligo has been established through these various mechanisms of action. While these treatments can be used as monotherapy, combining them with other methods can significantly enhance the recovery rate. For instance, Abdelghani et al. [1] demonstrated that combining PRP with CO_2_ fractional laser yielded better results than each modality alone, and was more effective than the combination of CO_2_ fractional laser with NB‐UVB. In a separate study by Afify et al. [2], a combined treatment of NB‐UVB, fractional CO_2_ laser, and PR was associated with the highest rate of improvement compared to groups receiving only CO_2_ laser and PRP, as well as those receiving CO_2_ fractional laser and NB‐UVB. However, no significant difference in recovery rates was observed among the three groups (*p* > 0.05).

The combination of 308‐nm excimer light and PRP has shown a significant therapeutic response and good patient tolerance [36]. Deng et al. [37] demonstrated significant differences between this combination and each treatment alone. Likewise, Ebadi et al. [4] found that combining 308‐nm excimer light with non‐cultured melanocyte‐keratinocyte transplantation resulted in significant therapeutic responses without serious complications. Elgarhy et al. [10] showed that combining non‐cultured melanocyte‐keratinocyte transplantation with NB‐UVB also led to significant improvement and good tolerance. Overall, it seems that combining excimer laser and NB‐UVB treatment with regenerative therapies is both safe and effective.

Additionally, injectable platelet‐rich fibrin (I‐PRF) is a second‐generation, fully autologous biomaterial that plays a significant role in regenerative medicine and the recovery of vitiligo lesions. It features a three‐dimensional fibrin mesh similar to a PRF clot while maintaining a fluid consistency akin to PRP. I‐PRF consists of platelets and their growth factors, along with type‐1 collagen and lymphocytes, as well as other essential growth factors [51, 58].

However, our risk of bias assessment revealed significant methodological concerns, with nearly half of the studies (48.84%) rated as high risk overall. Major issues included the randomization process (44.19% high risk) and deviations from intended interventions (62.79% high risk). While bias due to missing outcome data (62.79% low risk) and outcome measurement (95.35% low risk) was generally well managed, some concerns still remain. Encouragingly, all studies demonstrated low risk for selective reporting. These findings align with previous dermatology reviews, indicating the need for more rigorous study designs, transparent randomization methods, and strict intervention adherence to enhance research quality in vitiligo treatment.

## 5 Limitations

While regenerative treatments show promising potential for managing specific cases of vitiligo, it is crucial to acknowledge the high risk of bias present in the reviewed studies. This bias complicates our ability to accurately assess the efficacy and safety of these treatments. Furthermore, the lack of clearly validated outcome measures makes it challenging to draw definitive conclusions regarding their effectiveness. Many studies report positive results without using validated scores and often fail to examine critical aspects such as cosmetic acceptability, quality of life, treatment burden, or other patient‐reported outcome measures (PROMs).

## 6 Conclusion

Our systematic review concludes that regenerative medicine plays a significant role in managing vitiligo. Among these treatments, cell therapy—especially autologous non‐cultured melanocyte‐keratinocyte transplantation—and platelet‐rich plasma (PRP) injections have demonstrated particularly promising results. Our findings suggest that optimal treatment outcomes are achieved by combining regenerative medicine with other modalities, such as Excimer laser and NB‐UVB phototherapy.

It is essential to further evaluate these regenerative approaches against traditional therapies, particularly phototherapy, to gain a clearer understanding of their comparative effectiveness over the long term. Although existing studies highlight the immediate efficacy of regenerative methods, the absence of substantial long‐term follow‐up data limits our ability to assess their sustainability and overall impact on patient quality of life.

Therefore, we recommend designing additional clinical trials to explore new regenerative medicine approaches, such as stromal vascular fraction (SVF), exosomes, and stem cell‐conditioned medium for treating vitiligo. These studies should include robust comparisons with traditional treatment methods and incorporate long‐term follow‐up to better inform clinical practice and treatment strategies for this condition.

## Author Contributions

A.G. and A.J. designed the study. A.J. and A.P.M. wrote the paper. A.G. edited the manuscript. All authors have read and approved the content of the manuscript.

## Ethics Statement

The researchers were committed and adhered to the principles of the Helsinki Convention and the Ethics Committee of the Iran University of Medical Sciences in all stages.

## Consent

The authors obtained consent to publish. The current manuscript contains no individual person's data. Therefore, consent to publish is not applicable.

Consent to Participate: This project was approved by the Ethics Committee with the title: “A Systematic Review of Case Series and Clinical Trials Investigating Regenerative Medicine for the Treatment of Vitiligo”.

## Conflicts of Interest

The authors declare no conflicts of interest.

## Transparency Declaration

Authors declare that the manuscript is an honest, accurate, and transparent. No important aspect of the study is omitted.

## Data Availability

The data that support the findings of this study are available from the corresponding author upon reasonable request.
